# Rationally Designed α-Conotoxin Analogues Maintained Analgesia Activity and Weakened Side Effects

**DOI:** 10.3390/molecules24020337

**Published:** 2019-01-18

**Authors:** Chen Liu, Pengxiang Wu, He Zhu, Paolo Grieco, Ruihe Yu, Xinmei Gao, Guiyue Wu, Dong Wang, Hanmei Xu, Weiyan Qi

**Affiliations:** 1 State Key Laboratory of Natural Medicines, Ministry of Education, The Engineering Research Center of Synthetic Polypeptide Drug Discovery and Evaluation of Jiangsu Province, Department of Marine Pharmacy, China Pharmaceutical University, Nanjing 211198, China; hsdalan@163.com (C.L.); wuprocas@stu.cpu.edu.cn (P.W.); m18013927001@163.com (R.Y.); 15189806987@163.com (X.G.); cpu_yaodawgy@163.com (G.W.); ccbaby1229@163.com (D.W.); 2 Mason Laboratory, Department of Mechanical Engineering & Materials Science, Yale University, New Haven, CT 06520, USA; h.zhu@yale.com; 3 State key laboratory of mechanics and control of mechanical structures, Department of Aerocraft Design, College of Aerospace Engineering, Nanjing University of Aeronautics and Astronautics, Nanjing 210016, China; zhuhe0728@nuaa.edu.cn; 4 Department of Pharmacy, University of Naples Federico II, Naples, 49 80131 Via D. Montesano, Italy; paolo.grieco@unina.it

**Keywords:** conotoxin, nAChR, analgesia, side effects

## Abstract

A lack of specificity is restricting the further application of conotoxin from *Conus bullatus* (*BuIA*). In this study, an analogue library of *BuIA* was established and virtual screening was used, which identified high α7 nicotinic acetylcholine receptor (nAChR)-selectivity analogues. The analogues were synthesized and tested for their affinity to functional human α7 nAChR and for the regulation of intracellular calcium ion capacity in neurons. Immunofluorescence, flow cytometry, and patch clamp results showed that the analogues maintained their capacity for calcium regulation. The results of the hot-plate model and paclitaxel-induced peripheral neuropathy model indicated that, when compared with natural *BuIA*, the analgesia activities of the analogues in different models were maintained. To analyze the adverse effects and toxicity of *BuIA* and its analogues, the tail suspension test, forced swimming test, and open field test were used. The results showed that the safety and toxicity of the analogues were significantly better than *BuIA*. The analogues of *BuIA* with an appropriate and rational mutation showed high selectivity and maintained the regulation of Ca^2+^ capacity in neurons and activities of analgesia, whereas the analogues demonstrated that the adverse effects of natural α-conotoxins could be reduced.

## 1. Introduction

Cancer pain, chemotherapy-induced pain, and acute or chronic pain have become a Gordian knot in clinical practice. Prescription opioids are still the first-line treatments for analgesia, but their abuse has escalated rapidly, leading to a dramatic increase in cases of opioid dependence and overdose deaths [1]. As stated in a National Institute of Health (NIH) report, prescription opioids are the main category of medications presenting abuse liability; the number of prescriptions for oxycodone, hydrocodone, and other opioids have escalated from 76 million in 1991 to 207 million in 2013. The increased availability of prescribed opioids has been accompanied by dreadful consequences related to their abuse. Thus, an alternative therapeutic approach with non-opioid compounds is essential. Haylie reported a novel therapy based on a non-opioid mechanism that could effectively treat pain, with a disease-modifying action [2]. Conotoxins are the products of the Conus genus and are typically a range of cysteine-rich bioactive peptides [3]. Conotoxins, which target dozens of ion channels and receptors with high selectivity and affinity, can be treated as leading pharmacological compounds [4,5]. Currently, conotoxins are used for treating cardiogenic diseases and analgesia [6,7]. Ziconotide, ω-conotoxin *Conus magus* (MVIIA), is an approved drug that targets the calcium channel. Its mode of action proves that the calcium channel is a potential target for analgesia [8]. Although ziconotide is an approved analgesic drug, intrathecal injection should be used in clinical practice due to the blood-brain barrier.

The blood-brain barrier presents a challenge in the development of analgesic peptides. Nicotinic acetylcholine receptors (nAChRs), members of the ion channel family, play a vital role in modulating neuronal signaling transmission through the synaptic system at central and peripheral neurons [9]. Due to the high specificity and affinity for different nAChR subtypes, α-conotoxins have been deemed to be the most precise tools for nAChRs studies, and have shown effects in preclinical testing [10,11,12]. α9α10 nAChR had been identified and considered as a potential molecular target that can be used for analgesia. Pioneering work demonstrated that conotoxin Vc 1.1 has a strong analgesic activity, and it has entered clinical trials for the treatment of chronic pain [13,14]; however, human clinical trials were terminated due to the negative results of the phase II trials [15]. 

α7 nAChR can effectively regulate the permeability of calcium ions and modulate physiological or pathological mechanisms. It has a close relationship with calcium ions and is distributed in the presynaptic membrane and postsynaptic membrane [16]. When α7 nAChR in the presynaptic membrane is sensitized, it can accelerate calcium influx and enhance the fusion of the presynaptic membrane or vesicle [17]. Thus, the presynaptic membrane is depolarized, then the neural transmitters are released. In the postsynaptic membrane, α7 nAChR plays a vital role in the process of excitatory signal transduction and adjusting the delivery of gamma-aminobutyric acid (GABA). Gong et al. found that α7 nAChR was closely related to neuralgia [18]. α7 nAChR has a high conservation rate between species and is considered a potential candidate target for analgesia. The affinity of *BuIA* with α7 nAChR plays an important role in analgesia, but the capability of combining it with other types of nAChRs is the key to side effects and toxicity. Due to the lack of selectivity of *BuIA*, further applications of *BuIA* have been limited. Thus, in this work, *BuIA* was selected and some analogues were designed to enhance the selectivity of α7 nAChR. Then, the analogues were synthesized to evaluate the analgesic activity and the side effects.

## 2. Results

### 2.1. Virtual Screening and HPLC Chromatography of Analogues

**Figure 1:**
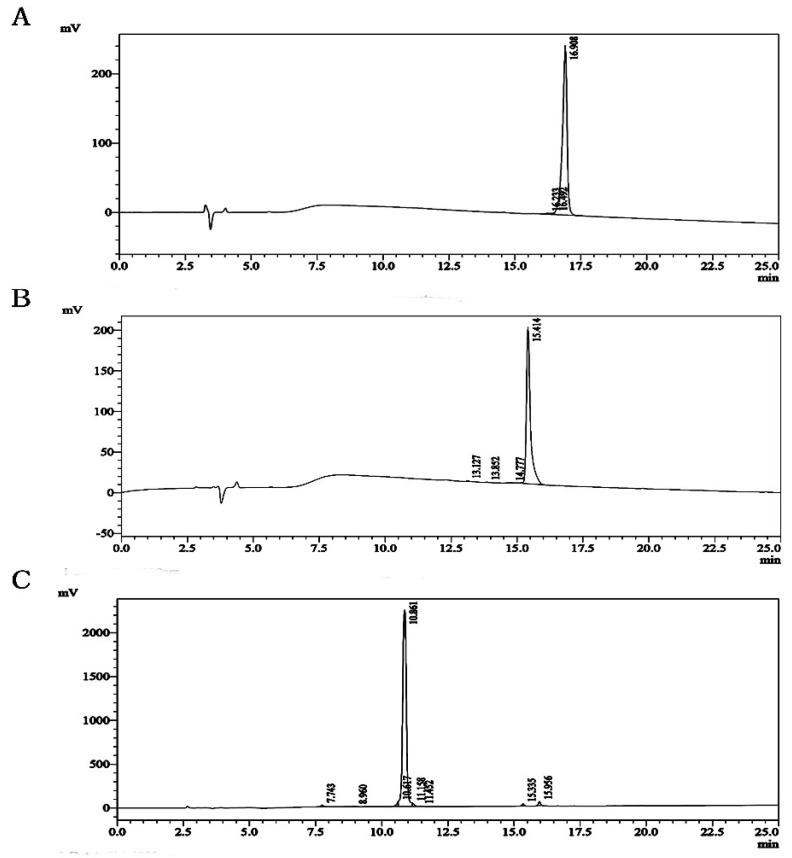
The design and the HPLC chromatography of (**A**) *cotx 2.1*, showing a purity of 98.826%; (**B**) of *cotx 2.13*, showing a purity of 99.215%; and (**C**) *cotx 1.1*, showing a purity of 95.573%.

Molecular Operating Environment (MOE) software (Chemical Computing Group, Quebec, Canada) (molecular modeling and simulations) was used to analyze the relationship between *Conus bullatus (BuIA)* and soluble acetylcholine receptor (PDB ID: 4EZ1). *BuIA* contains two disulfide bonds CysI–CysIII and CysII–CysIV and maintains a stable α-helix structure, which plays an important role in the interaction between *BuIA* and nAChR. Exploring the structural characteristics and disulfide connectivity of this complex would provide more information about α-conotoxin–*BuIA* interactions. Therefore, we designed a *BuIA* analogue database and performed virtual screening, where the interaction sites between two subunits and analogues were selected according to previous work [19,20,21,22] and described in the Methods section. The results showed that the affinity between α7 nAChR subunits with *cotx 2.1* and *cotx 2.13* was higher than *BuIA*, and the affinity between α3, α6, β2, or β4 nAChRs with *cotx 2.1* and *cotx 2.13* weakened. However, the affinities between all the nAChRs with Loop1 analogues weakened (Table 1). Therefore, in this work, *cotx 1.1* (GCSSTPPC, Cys I-Cys II) was selected as the Loop1 analogue. Then, *cotx 2.1*, *cotx 2.13*, and *cotx 1.1* were designed and synthesized. High performance liquid chromatography (HPLC) profiles showed that the purities of the *cotx 2.1*, *cotx 2.13*, and *cotx 1.1* that we synthesized were higher than 95%, and that the compounds therefore suitable for use in subsequent experiments (Figure 1). The mass spectrometry (MS) results showed that the molecular weights (MWs) of *cotx 2.1*, *cotx 2.13*, and *cotx 1.1* were 1281.46, 1293.52, and 747.85, respectively (Figure S1). In proton nuclear magnetic resonance (^1^H NMR) and carbon-13 (^13^C) NMR spectra, all of the purified analogues had well dispersed signals, which means that in the solution, the analogues adopted ordered structures (Figures S2–S4).

### 2.2. Affinity and Activity of BuIA and Analogues

**Figure 2:**
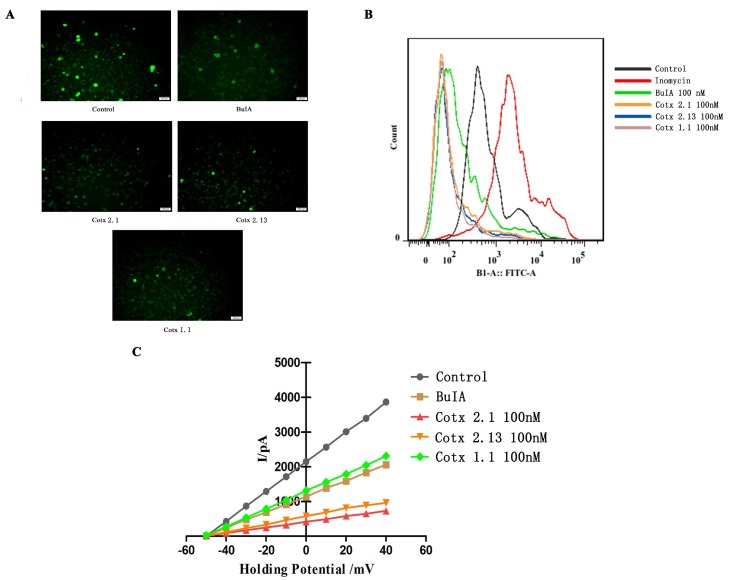
Activity of *BuIA* and its analogues on Ca^2+^ levels in the dorsal root ganglion (DRG) neuron cells: (**A**) immunofluorescence images of calcium in the DRG neuron cells that were treated with *BuIA* and its analogues: *BuIA* and all of the analogues significantly weakened the fluorescence intensity of intracellular calcium ion staining; (**B**) flow cytometry results of calcium in the DRG neuron cells that were incubated with *BuIA* and its analogues: *BuIA* and all of the analogues significantly reduced the calcium concentration in DRG cells; (**C**) the results of the calcium channel state in the DRG neuron cells. The patch clamp was used and the cells were treated with *BuIA* and its analogues: *BuIA* and all of the analogues inhibited the calcium channel on the DRG cell surface. The inhibitions of *cotx 2.1* and *cotx 2.13* were significantly higher than *BuIA* and *cotx 1.1*, and the differences between *BuIA* groups and *cotx 1.1* groups had no statistical significance.

In this work, we analyzed the affinities of *BuIA* and its analogues with human α7 nAChR. Compared with the *BuIA*, the affinity between human α7 nAChR and *cotx 2.1* or *cotx 2.13* was significantly enhanced (*p* < 0.05). Although the affinity between the human α7 nAChR and *cotx 1.1* was weaker, the affinity of *BuIA* and *cotx 1.1* was in the same order of magnitude (Table 2 and Table S5). Then, the calcium levels in the dorsal root ganglion (DRG) neuron cells were investigated by immunofluorescence and flow cytometry. The results showed that after being treated with *BuIA* and its analogues, the calcium levels in Dorsal Root Ganglion (DRG) neuron cells decreased (Figure 2A,B). As per a previous study [23], the patch clamp was used to detect the influence of *BuIA* and its analogues on Ca^2+^ in DRG cells. The results showed that *BuIA* and its analogues could induce calcium channel closure. The inhibitions of *cotx 2.1* and *cotx 2.13* were stronger than *BuIA* and *cotx 1.1*, although the inhibitions of *BuIA* and *cotx 1.1* did not show obvious differences (Figure 2C).

### 2.3. Analgesic Activity of BuIA and Analogues In Vivo

**Figure 3:**
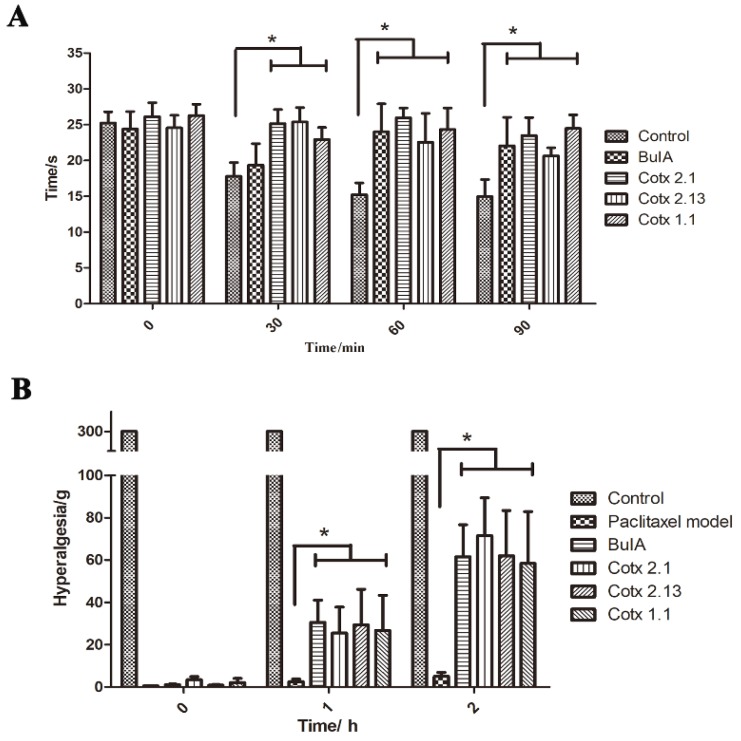
Analgesic activity of *BuIA* and analogues. Bar graphs represent the mean ± SEM,* *p* < 0.05: (**A**) Hot-plate test model: control group treated with saline. The *BuIA* group was treated with 1.5 mg/kg *BuIA*, the *cotx 2.1* group was treated with 1 mg/kg *cotx 2.1*, the *cotx 2.13* group was treated with 1.5 mg/kg *cotx 2.13*, and the *cotx 1.1* group was treated with 2 mg/kg *cotx 1.1*. Variance analysis showed that the licking times of the *BuIA* and analogue groups were significantly higher than the control group (*F* = 5.608, *p* < 0.05). (**B**) Paclitaxel-induced peripheral neuropathy model: the control group was treated with saline. The paclitaxel model, with the *BuIA* and analogue groups intraperitoneally injected with paclitaxel (10 mg/kg), was used to establish the models. After 24 h, the paclitaxel model group was treated with saline, the *BuIA* group was treated with 1.5 mg/kg *BuIA*, the *cotx 2.1* group was treated with 1 mg/kg *cotx 2.1*, the *cotx 2.13* group was treated with 1.5 mg/kg *cotx 2.13*, and the *cotx 1.1* group was treated with 2 mg/kg *cotx 1.1*. Variance analysis showed that the hyperalgesia of *BuIA* and the analogue groups were significantly higher than the control group. However, the differences between the *BuIA* and analogue groups were not statistically significant (*F* = 3.849, *p* < 0.05).

The analgesic activities of *BuIA* and its analogues were evaluated using the hot-plate test model and the paclitaxel-induced peripheral neuropathy model. In the hot-plate test model, when compared to the control group after injection, the licking time of *BuIA* and the analogue groups significantly expanded (*p* < 0.05), but the difference between the *BuIA* group and analogue groups was not statistically significant (*p* > 0.05). Further analysis showed that the efficacy times of *cotx 2.1* and *cotx 2.13* were quicker than *BuIA* and *cotx 1.1* (Figure 3A and Figure S6). When the paclitaxel-induced peripheral neuropathy model was applied, the hyperalgesias of *BuIA* and analogue groups improved significantly (*p* < 0.05). There were no significant differences within the *BuIA* group and the analogue groups (*p* > 0.05) (Figure 3B). 

### 2.4. Adverse Reactions to BuIA and Analogues

**Figure 4:**
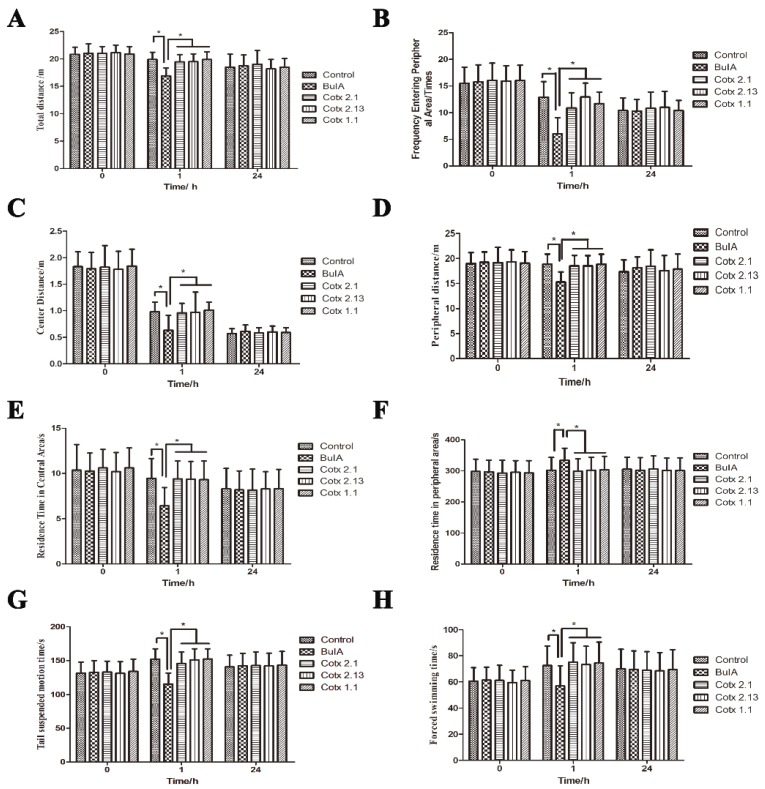
Adverse reactions of *BuIA* and analogues. Bar graphs represent the mean value ± SEM, * *p* < 0.05. (**A**) Open field test—total distances: the total distances of the analogue groups and control group had no significant differences (*p* > 0.05), but the *BuIA* group was significantly lower than the other groups (*p* < 0.05); (**B**) open field test—frequency entering peripheral area: the frequency entering the peripheral area of the analogue groups and control group showed no significant differences (*p* > 0.05), but the *BuIA* group was significantly lower than the other groups (*p* < 0.05); (**C**) open field test—center distance: the center distance of the analogue groups and control group had no significant differences (*p* > 0.05), but the *BuIA* group was significantly lower than the other groups (*p* < 0.05); (**D**) open field test—peripheral distance: the peripheral distance of the analogue groups and control group had no significant differences (*p* > 0.05), but the *BuIA* group was significantly lower than the other groups (*p* < 0.05); (**E**) open field test—residence time in central area: the residence time in the central area of the analogue groups and control group had no significant differences (*p* > 0.05), but the *BuIA* group was significantly lower than the other groups (*p* < 0.05); (**F**) open field test—residence time in peripheral area: the residence time in the peripheral area of the analogue groups and control group had no significant differences (*p* > 0.05), but the *BuIA* group was significantly higher than the other groups (*p* < 0.05); (**G**) the tails suspension test—tail suspended motion times: the tail suspended motion times of the analogue groups and control group demonstrated no significant differences (*p* > 0.05), but the *BuIA* group was significantly lower than the other groups (*p* < 0.05); (**H**) forced swimming test—forced swimming times: the forced swimming times of the analogue groups and control group had no significant differences (*p* > 0.05), but the *BuIA* group was significantly lower than the other groups (*p* < 0.05).

There is a close relationship between α7 nAChR and autonomic movement, so the open field test, tails suspension test, and forced swimming test were used to analyze the changes in autonomic movement after the mice were treated with *BuIA* and the analogues. All the results showed that *cotx 2.1, cotx 2.13*, and *cotx 1.1* did not affect the autonomic movement of mice (Figure 4). However, when compared with the control group, *BuIA* weakened the autonomous movement of mice significantly (*p* < 0.05). Twenty-four hours later, there were no significant differences between all of the groups (*p* > 0.05) (Figure 4).

## 3. Discussion

Ion channels are important analgesic targets. Ziconotide, with low addiction potential and good effect, has been approved for clinical use, although intrathecal administration has limited its clinical application due to the blood–brain barrier. Therefore, peripheral ion channels are receiving increased attention. As reviewed by Vink and Alewood [24], α7 and α9α10 are the peripheral targets for inflammatory and neuropathic pain. Azam [25] described the activity of α-conotoxin *BuIA* on 10 different nAChRs with genetic mutations and the results showed that *BuIA* can block different types of nAChRs. Normally, compounds with low specificity likely produce more side effects. Therefore, we established an analogue library based on *BuIA* to find an analogue that specifically targets α7 nAChR. In this work, α7 nAChR was selected as the peripheral analgesia target, so α-conotoxin *BuIA*, which can target α7 nAChR and other types of nAChRs, was selected to design analogues with high α7 nAChR specificity.

Yu et al. [23] found that the deletion of a disulfide bridge in α-conotoxins did not strongly influence its overall structure and activity, but they added another linker to maintain the binary ring structure. The question of whether the deletion of the disulfide bridge had an effect on specificity needed to be confirmed. Therefore, we designed a single disulfide bridge mutant library without adding any linker and analyzed whether analogues with higher specificity could be obtained. As Cuny [19] stated, Loop2 is an important factor in determining the selectivity of α-conotoxin. Therefore, in this work, we analyzed the complex structure of *BuIA* and acetylcholine binding protein and treated *BuIA*-Cys8 as the component of Loop2. Neutral or hydrophilic amino acids were used to replace *BuIA*-Cys8, according to the characteristics of Cys. Although the conotoxin selectivity was attributable to Loop 2, the sequences of Loop 1 and Loop 2 have high repeatability. Servents’ [26] research showed that the key residue for the inhibition of nAChRs by α-conotoxin ImI was the Asp-Arg-Pro motif in the overlapping areas of Loop1 and Loop2. Therefore, in this work, we wanted to confirm whether Loop1 could enhance selectivity, so alterations to Loop 1 were performed. The results showed that that the analogues of Loop1 could not significantly improve the selectivity of α7 nAChR, but that the affinity to other receptor subtypes was diluted. Therefore, we speculated that this was the reason why *cotx 1.1* would have similar activity to Loop2 analogues at high dosages. Next, we wanted to obtain the analogues that had a strong affinity with α7 nAChR and maintain the affinity between the other subtypes of nAChRs. The virtual screening results showed that *cotx 2.1* and *cotx 2.13* could match the requirements, but all of the Loop1 analogues did not enhance their affinity with any subtype of nAChR. Therefore, the analogue with a high affinity for α7 nAChR and the same mutation pattern as *cotx 2.1* was selected for further research.

Subsequently, *cotx 2.1*, *cotx 2.13*, and *cotx 1.1* were synthetized with solid phase peptide synthesis (SPPS) and the purity met all requirements. The ^1^H NMR spectra showed that all of the purified analogues had well dispersed signals, which meant that the analogues had adopted ordered structures in the solution. We investigated the affinity between *BuIA* and its analogues and human α7 nAChR by microscale thermophoresis analysis [27,28]; the affinity between the functional α7 nAChR protein and *cotx 2.1* and *cotx 2.13* was enhanced. However, the affinity between the α7 nAChR protein and *cotx 1.1* decreased. Due to the changes in structure and sequence, the orientation of cysteine changed, causing the core sequences to be more exposed to the combination of *BuIA* analogues, and α7 nAChR was strengthened. *cotx 1.1*, lacking one loop structure, showed that the connection with α7 nAChR weakened.

A Fluo-4 AM (AAT Bioquest Inc, Sunnyvale, CA, USA) probe was utilized to mark the calcium ion in rat DRG = neurons cells. Flow cytometry and fluorescence microscope analysis showed that *BuIA* and its analogues could significantly reduce the calcium ion levels in the DRG cells, but the inhibition of *BuIA* and its analogues showed no significant differences (*p* > 0.05). The patch clamp results showed that *BuIA* and all of its analogues could limit the circulation of the calcium flux. Based on these results, the hot-plate test model and paclitaxel-induced peripheral neuropathy model were used to assess the analgesic activity of *BuIA* and its analogues.

Side effects and the safety of the analgesic compounds are vital to drug development [29], so we also tested the side effects and the toxicity of *BuIA* and its analogues. The toxicity of all compounds were tested using Caenorhabditis elegans (*C. elegans*). And the analogues could maintain the heat resistance threshold and hyperalgesia of *BuIA*, whereas the side effects and the toxicity (Supplementary Material Figure S7) of the analogues were weaker compared with *BuIA*. Low specificity is the key to causing sides effects. So, effectively enhancing the selectivity is one of the important ways to reduce side effects. And this is the reason that the *cotx 2.1* and *cotx 2.13* could maintain the analgesia activity and weaken the side effects.

In this work, we synthesized single disulfide analogues, *cotx 2.1*, *cotx 2.13*, and *cotx 1.1*, without changing the pairing models of disulfide (CysI-CysIII and CysII-CysIV). Compared with the natural *BuIA*, the structures of these analogues were simplified, which improved the efficiency of their synthesis with better quality, as well as being faster and cheaper. As in previous studies, single disulfide conotoxin analogues could be developed into new analgesic leading compounds [30]. In our work, the results showed that the *cotx 2.1* and *cotx 2.13* mutations of *BuIA* could enhance selectivity with α7 nAChR and adjust the calcium flow into the DRG neuron cells. All of the analogues maintained the analgesic activity of *BuIA*, and the analogues also had low toxicity and reduced adverse reactions in vivo. This suggests that the deletion of the disulfide bridge of α-conotoxin not only maintained activity, but also enhanced the selectivity and obtained highly specific targeted analogues by the appropriate mutation methods. However, α7 nAChR is closely related to the treatment of inflammatory pain, which is highly correlated with astrocytes. Therefore, whether the analogues combined with drugs that regulate the astrocyte can enhance analgesic activity remains to be studied. Intracranial α7 nAChR is closely related to depression and Parkinson’s disease. Next, we will modify the structure of *cotx 2.1* or *cotx 2.13* via side chain modifications, and use the strapped peptide strategy to cross the blood–brain barrier and analyze the potential for the treatment of other diseases such as Parkinson’s disease.

## 4. Materials and Methods

### 4.1. Analogue Design and Siftings

Based on the crystal structure of acetylcholine binding protein (AChBP) in complex with α-conotoxin *BuIA* (PDB ID:4EZ1), *BuIA*-Cys8 plays a vital role in binding to the AChBP, which is also a key in maintaining the structure of *BuIA*. Loop2 is an important factor determining the selectivity of α-conotoxin [19]. Therefore, in this work, we treated *BuIA*-Cys8 as the component of Loop2. According to the characteristics of Cys, neutral amino acids or hydrophilic amino acids were used to replace *BuIA*-Cys8 and *BuIA*-Cys2. Lys, Arg, and His (basic and hydrophilic amino acids) were used instead of *BuIA*-Cys2, and *BuIA*-Gly1 was deleted, while *BuIA*-Cys8 was replaced with Asp, Gln, and Ser (neutral and hydrophilic amino acids). The analogues of Loop1 were established, *BuIA*-Cys3 was replaced with neutral amino acids or hydrophilic amino acids, and the amino acids from *BuIA*-Ala9 to *BuIA*-Cys13 were deleted. As Azam [25] demonstrated, *BuIA* can combine with α3, β2, β4, α6, and α7 nAChR subunits, so the α3 (PDB ID:5T90), β2 (PDB ID: 5KXI), β4 (PDB ID:5T90), α6 (PDB ID:2BYP), and α7 (PDB ID:3SQ6) structures were download from PDB. According to previous research, the docking sites (α3 [20], β2 [19], β4 [19], α6 [21] and α7 [22]) which were between two subunits were selected; then, the Molecular Operating Environment (MOE) 2009 software (2009, Montreal, Canada) was used for docking, and the scoring functions were Triangle Matcher and Forcefield.

### 4.2. Peptide Preparation

*BuIA* was purchased from GenScript (GenScript, Nanjing, China), whereas GL Biochem (GL Biochem, Shanghai, Chinae supplied H-Cys(Trt)-2-CTC resin and amino acids. The fluorenylmethyloxycarbonyl (Fmoc) solid phase peptide synthesis (SPPS) methods were used to synthesize all the analogues using the Focus XC system (AAPPTec, Louisville, KY, USA). *N*,*N*′-diisopropylcardodiimide (DIC) and 1-hydroxbenzotriazole (HOBt) were applied as a peptide coupling reagent and piperidine (PIP) was chosen as a deprotection reagent in the synthesis process. In the process of cleavage, 90% trifluoroacetic acid (TFA) solution was selected as a cleavage buffer, and ether was used as a settling agent. Peptides were dissolved in acetonitrile and aqueous buffer (pH 8.0, 1 mg/mL) overnight for oxidative cyclization. An Alltima^TM^ C18 column (4.6 × 250 nm) (Agilent Technologies Inc, Santa Clara, CA, USA) was used to purify the crude peptide with eluent monitor at 220 nm. When the HPLC was operated, 0.065% trifluoroacetic acid in 100% water was prepared as buffer A, and buffer B was 0.05% trifluoroacetic acid in 100% acetonitrile. The flow speed was set to 1 mL/min. The same conditions were used in HPLC-MS (LCMS 2000, SHIMADZU, Kyoto, Japan) analysis to confirm the purity and molecular mass of the analogues. All analogues were dissolved in D_2_O (J&K Scientific, Beijing, China), then a Bruker AVANCE AV-500 (Bruker Daltonics Inc., San Diego, CA, USA) was used to record the spectra. The NMR experiments included ^13^C-NMR and ^1^H-NMR.

### 4.3. Microscale Thermophoresis Analysis

Besides the radioligand assay [31,32] and surface plasmon resonance (SPR) [33], microscale-thermophoresis (MST) is another technique used to analyze the interaction between the receptor and ligand based on the thermal motion of biomolecules [34]. Human functional receptor protein (α7 nAChR) was purchased from Cloud-Clone Corp (Cloud-Clone Corp, Houston, TX, USA); this was first dissolved into a buffer solution that did not contain primary amine compounds. The buffer solution was exchanged through a mixed column A. Then, Cy5 fluorescent dye (Ruixin Biological Technology, Xi’an, China) was added to stain the recombinant protein and purify the labeled protein through the purified column B. Finally, the analogues were diluted by a certain proportion and mixed with the labeled protein one to one, incubated at room temperature for 5 min, and the relevant instrument parameters were set. Monolith NT.115 (NanoTemper, Munich, Germany) and NT-Analysis software (NanoTemper, Munich CITY, Germany) were used to record and analyze the data.

### 4.4. Dorsal Root Ganglion Neuron Cell Preparation

The 120–160 g Wistar rats were killed by cervical dislocation and the ganglia was enzymatically dissociated to obtain the dorsal root ganglion (DRG) neuron cells. The operation procedures were investigated and approved by the Animal Ethics Committee of China Pharmaceutical University (SYXK(SU)2016-0011). Normal feeding occurred for at least three days for the animals to adapt to the environment, and we ensured that the mice had a free water supply during the experiment. As described in the literature [23,35], the ganglia were rinsed in ice-cold Hank’s Balanced Salt Solution (HBSS) (Life Technologies, Carlsbad, CA, USA) and incubated in HBSS buffer containing 1.5 mg/mL type 2 collagenase (Sigma-Aldrich Inc., Milwaukee, WA, USA) at 37 °C for 30 min. Then, Dulbecco’s Modified Eagle’s Medium (DMED) (Gibco, Carlsbad, CA, USA), supplemented with 1% penicillin/streptomycin and 10% fetal calf fetal (Biological Industries, Kibbutz Beit Haemek, Israel) was used to rinse the ganglia three times and triturated with Pasteur pipettes. The DRG neuron cells were plated on a poly-D-lysine (Beyotime, Shanghai, China)/laminin (Sigma-Aldrich, Milwaukee, USA)-coated plate (Thermo Fisher, Massachusetts, MA, USA), incubated at 37 °C in 5% CO_2_ at a relative humidity of 95%. All cells were used within 48 h.

### 4.5. Fluorescence Imaging Analysis

The fluorescence labeling technique was used to combine the studied protein with some fluorescent substances, and uses its fluorescence properties to provide a range of information about the protein [36]. Fluo-4 acetyloxymethyl (AM) is an acetyl methyl ester derivative of Fluo-4 that can be cleaved by intracellular esterase to Fluo-4. Fluo-4 binds to calcium ions to produce strong fluorescence with a maximum excitation wavelength of 494 nm and a maximum emission wavelength of 516 nm [37]. A certain number of analogues and natural α-conotoxins were accurately measured. Sample solutions with a concentration of 100 nM were prepared using serum-free DMEM medium with the DRG neuron cells as a culture object. After 4 h, the cells were rinsed with phosphate buffer saline (PBS) several times, then 2 μM of Fluo-4 AM calcium ion fluorescent dye (AAT Bioquest Inc, Sunnyvale, CA, USA) was added to each hole, and the cells were incubated for 30 min at room temperature. Results were observed and recorded under a fluorescence microscope (OLYMPUS IX53, Tokyo, Japan), where the fluorescence imaging analysis was repeated three times.

### 4.6. Flow Cytometer Analysis

Flow detection technology is a multi-parameter, rapid quantitative analysis and sorting technique for cells and biological particles in rapid linear flow. An analogue and *BuIA* were accurately measured. Sample solutions with a concentration of 100 nM were prepared using serum-free DMEM medium and using the DRG neuron cells as a culture object. After 4 h, the cells were rinsed with PBS several times, and 500 μL of Fluo-4 AM calcium ion fluorescent dye and 1 μg/mL of calcium ionomycin were added to each hole. Then, the cells were incubated at room temperature for 30 min. Two independent validations were performed for flow cytometer analysis, then the samples were detected by a MACSQuant flow cytometer (Miltenyi Biotec, Bergisch Gladbach, Germany) and FlowJo 7.6 (FlowJo LLC, Ashland, AL, USA) was used to analyze the data.

### 4.7. Patch Clamp Electrophysiology

The patch clamp is a membrane that is touched by a micro-glass electrode, which is connected by resistance above a gigabit; then, the ion current (pA level) of the ion channel on the membrane is detected and recorded [38]. The analogues and *BuIA* were measured. Sample solutions were prepared by using serum-free DMEM medium and DRG nerve cells as the culture object. The whole-cell configuration of the patch clamp technique with a QPatch-16 workstation (Sophion, Ballerup, Denmark) was used to record the membrane currents in the DRG neurons. The external recording solution contained the following: 10 mM 4-(2-hydroxyethyl)-1-piperazineethanesulfonic acid (HEPES) buffer, 10 mM D-glucose, 150 mM tetraethylammonium-Cl, and 2 mM BaCl_2_ at pH 7.3. The internal solution, which contained 10 mM HEPES, 1 mM MgCl_2_, 140 mM CsCl, 0.1 mM Na-GTP, 4 mM MgATP, and 5 mM 1,2-bis(O-aminophenoxy) ethane-*N*,*N*,*N*′,*N*′-tetraacetic acid tetracesium salt-Cs4 at pH 7.3, was used to the fill the fire-polished record electrodes. During recording, a gravity-fed perfusion system was used to perfuse the DRG neurons with external recording at a flow rate of 1 mL/min. The currents of the DRG neuron cells of each group were detected with the QPatch-16 workstation (Sophion, Ballerup, Denmark) and data were processed with supporting software. In order to verify the reliability of the results, we also carried out two repeated experiments.

### 4.8. Animals

Female Kunming mice (20–25 g) and Wistar rats (120–160 g) used in this study were obtained from the Experimental Animal Center of Qinglong Mountain in Nanjing, China. The animals were maintained until 7–8 weeks old. Normal feeding occurred for at least three days for the animals to adapt to the environment, and we ensured that the mice had a free water supply during the experiment. All the mice were cared for according to the Provisions and General Recommendation of Chinese Experimental Animals Administration Legislation (SYXK(SU)2016-0011).

#### 4.8.1. Hot-Plate model Test

The mice were placed on an enclosed hot plate (Yuyan instruments, Shanghai, China). The plate temperature was set at 55 ± 0.5 °C. The time (hot plate pain threshold) was recorded and the mice were removed from the enclosed hot-plate when the mice licked a hind paw. The mice were first screened for pain threshold and were retained between 10–30 s. The screened mice were divided into the blank group, *BuIA* group, and the analogues groups with 12 mice in each group. The control group was treated with saline, the *BuIA* group was treated with 1.5 mg/kg *BuIA*, the *cotx 2.1* group was treated with 1 mg/kg *cotx 2.1*, the *cotx 2.13* group was treated with 1.5 mg/kg *cotx 2.13*, and the *cotx 1.1* group was treated with 2 mg/kg *cotx 1.1*. According to the group, mice in each group were intraperitoneally injected with physiological saline, *BuIA*, or the analogues. After 0, 30, 60, and 90 min, the hot plate was used and the hot plate pain threshold was recorded for each group of mice. 

#### 4.8.2. Paclitaxel-Induced Peripheral Neuropathy Model

The mice were divided into a blank group, model group, *BuIA* group, and analogues groups with 12 mice in each group. Then, the mice in the model group, *BuIA* group, and analogues groups were treated with 10 mg/kg paclitaxel (Biopurify, Chengdu, China) with intraperitoneal injection. After 24 h, according to the group, the mice in each group were intraperitoneally injected with physiological saline. The control group was treated with saline, the *BuIA* group was treated with 1.5 mg/kg *BuIA*, the *cotx 2.1* group was treated with 1 mg/kg *cotx 2.1*, the *cotx 2.13* group was treated with 1.5 mg/kg *cotx 2.13*, and the *cotx 1.1* group was treated with 2 mg/kg *cotx 1.1*. After 0 h, 1 h, and 2 h, the Von Frey Hairs were used to test the hyperalgesia. Yuyan Instruments (Yuyan Instruments, Shanghai, China) supplied the hot plate analgesia test meter and the Von Frey Hairs instrument was purchased from North Coast Medical (North Coast Medical Inc., Morgan Hill, CA, USA).

### 4.9. Detection of Side Reaction Activity

#### 4.9.1. Tail Suspension Test

The mice were randomly divided into a blank group, *BuIA* groups, and analogues groups with 10 mice in each group. Mice in each group were intraperitoneally injected with physiological saline, *BuIA,* or the analogues. The control group was treated with saline; and the *BuIA* group, *cotx 2.1* group, *cotx 2.13* group, and *cotx 1.1* group were treated with 5 mg/kg *BuIA*, *cotx 2.1*, *cotx 2.13*, or *cotx 1.1*, respectively. The mice were kept motionless for a while after being hung, and the inactivity time within 5 min was recorded [39,40].

#### 4.9.2. Forced Swimming Test

The mice were randomly divided into a blank group, *BuIA* groups, and analogues groups with 10 mice in each group. Mice in each group were intraperitoneally injected with physiological saline, *BuIA*, or the analogues, respectively. The control group was treated with saline, and the *BuIA* group, *cotx 2.1* group, *cotx 2.13* group, and *cotx 1.1* group were treated with 5 mg/kg *BuIA*, *cotx 2.1*, *cotx 2.13* or *cotx 1.1*, respectively. The mice were kept motionless for a while in the swimming state and the inactivity time in 5 min was recorded [40].

#### 4.9.3. Open Field Test

The mice were randomly divided into a blank group, *BuIA* groups, and analogues groups with 10 mice in each group. Mice in each group were intraperitoneally injected with physiological saline, *BuIA,* or the analogues, respectively. The control group was treated with saline and the *BuIA* group, *cotx 2.1* group, *cotx 2.13* group, and *cotx 1.1* group were treated with 5 mg/kg *BuIA*, *cotx 2.1*, *cotx 2.13* and *cotx 1.1*, respectively. The behavioral indicators (total distances, frequency entering peripheral area, center distance, peripheral distance, residence time in central area, residence time in peripheral area) of mice in the open field box in 5 min were observed and recorded [41].

### 4.10. Data Analysis

All data processing was conducted using special software, and the details are described in the materials and methods section, whereas the NMR spectroscopy data were analyzed with MestReNova (Mestrelab Research SL, Santiago de Compostela, Spain). The measurement data were analyzed by GraphPad Prism 5 (GraphPad software Inc., San Diego, CA, USA), then a two-way ANOVA was used to test the data differences between the groups.

## 5. Conclusions

In conclusion, we screened and synthesized novel *BuIA* analogues that targeted α7 nAChR specificity based on the *BuIA* analogue library. Although we simplified the structure of conotoxin *BuIA* by mutating the amino acid and deleting a disulfide bond, all of the analogues analgesic activities could improve the safety and reduce the incidence of adverse reactions to autonomic movement. However, research on the further possibility of *cotx 2.1* or *cotx 2.13* for the treatment of diseases such as Parkinson’s disease needs to be undertaken to improve its potential as an analgesic drug. Finally, the pharmacokinetics and preparation are obstacles in the next stage.

## 6. Patents

Xu Hanmei, Qi Weiyan, Liu Chen, and Wu Pengxiang. The peptide with analgesic activity and its application [P]. Jiangsu: CN107759665A, 2018-03-06.

## Figures and Tables

**Table 1:** The scores of *Conus bullatus (BuIA)* and analogues.

Number	Sequence	α3	α6	β2	β4	α7
*BuIA*	GCCSTPPCAVLYC (CysI–CysIII, CysII–CysVI)	−6.657	−5.032	−6.866	−5.485	−7.136
Loop2						
*cotx 2.1*	GSCSTPPSAVLYC (CysI–CysII)	−6.531	−4.783	−6.395	−4.544	−7.946
*cotx 2.2*	GDCSTPPDAVLYC (CysI–CysII)	−6.770	−5.060	−6.884	−5.377	−7.636
*cotx 2.3*	GQCSTPPQAVLYC (CysI–CysII)	−6.265	−4.799	−7.536	−4.297	−8.239
*cotx 2.4*	GMCSTPPMAVLYC (CysI–-CysII)	−6.878	−4.652	−7.407	−4.472	−8.766
*cotx 2.5*	GWCSTPPWAVLYC (CysI–CysII)	−7.265	−7.348	−7.333	−6.083	−8.073
*cotx 2.6*	GYCSTPPYAVLYC (CysI–CysII)	−7.106	−5.128	−7.123	−6.265	−7.541
*cotx 2.7*	GTCSTPPTAVLYC (CysI–CysII)	−6.259	−4.150	−7.186	−4.711	−7.857
*cotx 2.8*	GECSTPPEAVLYC (CysI–CysII)	−6.605	−3.883	−6.611	−4.528	−7.084
*cotx 2.9*	GHCSTPPHAVLYC (CysI–CysII)	−6.995	−7.399	−8.029	−5.695	−8.570
*cotx 2.10*	GKCSTPPKAVLYC (CysI–CysII)	−7.151	−5.698	−7.412	−4.759	−8.167
*cotx 2.11*	GRCSTPPRAVLYC (CysI–CysII)	−7.315	−5.643	−7.302	−5.565	−8.906
*cotx 2.12*	KCSTPPSAVLYC (CysI–CysII)	−7.267	−5.625	−6.204	−5.173	−7.719
*cotx 2.13*	KCSTPPDAVLYC (CysI–CysII)	−6.358	−4.509	−6.406	−5.363	−7.190
*cotx 2.14*	KCSTPPQAVLYC (CysI–CysII)	−6.448	−6.270	−6.617	−4.347	−7.755
*cotx 2.15*	HCSTPPSAVLYC (CysI–CysII)	−6.203	−5.498	−6.424	−4.907	−7.241
*cotx 2.16*	HCSTPPDAVLYC (CysI–CysII)	−6.451	−5.757	−6.406	−5.069	−7.774
*cotx 2.17*	HCSTPPQAVLYC (CysI–CysII)	−7.002	−6.143	−6.850	−4.313	−7.781
*cotx 2.18*	RCSTPPSAVLYC (CysI–CysII)	−7.177	−4.840	−6.789	−4.531	−8.072
*cotx 2.19*	RCSTPPDAVLYC (CysI–CysII)	−6.930	Neg	Neg	−5.294	Neg
*cotx 2.20*	RCSTPPQAVLYC (CysI–CysII)	−7.354	Neg	Neg	−5.259	Neg
Loop1						
*cotx 1.1*	GCSSTPPC (CysI–CysII)	−5.834	−4.028	−5.969	−3.186	−6.462
*cotx 1.2*	GCDSTPPC (CysI–CysII)	−5.983	−4.161	−5.718	−3.390	−6.376
*cotx 1.3*	GCQSTPPC (CysI–CysII)	−5.755	−6.275	−5.633	−3.289	−6.651
*cotx 1.4*	GCWSTPPC (CysI–CysII)	−6.281	−6.187	−5.403	−4.900	−6.966
*cotx 1.5*	GCYSTPPC (CysI–CysII)	−6.133	−4.823	−5.710	−5.271	−6.670
*cotx 1.6*	GCMSTPPC (CysI–CysII)	−6.233	−6.329	−5.454	−3.633	−6.392
*cotx 1.7*	GCTSTPPC (CysI–CysII)	−6.353	−4.967	−5.938	−3.553	−6.211
*cotx 1.8*	GCESTPPC (CysI–CysII)	−6.151	−3.414	−5.640	−3.669	−6.249
*cotx 1.9*	GCHSTPPC (CysI-CysII)	−6.045	−6.565	−5.506	−3.648	−6.010
*cotx 1.10*	GCKSTPPC (CysI–CysII)	−5.956	−4.409	−5.532	−3.282	−6.450
*cotx 1.11*	GCRSTPPC (CysI–CysII)	−6.373	−6.403	−5.770	−4.116	−6.370

**Table 2:** The affinity of *BuIA* or its analogues with functional α7 nicotinic acetylcholine receptor (nAChR).

Peptide	EC_50_ (nM)
*BuIA*	19.7 ± 2.85
*cotx 2.1*	12.3 ± 1.07
*cotx 2.13*	15.4 ± 1.6
*cotx 1.1*	26.6 ± 4.8

## Supplementary Materials

The Supplementary Materials are available online. Figure S1: Determination of the MS (Mass Spectrometer); Figure S2: Determination of the NMR of cotx 2.1; Figure S3: Determination of the NMR of cotx 2.13; Figure S4: Determination of the NMR of cotx 1.1; Figure S5: The Microscale Thermophoresis Analysis (MST) curves; Figure S6. Analgesic activity of BuIA and analogues; Figure S7 Acute and chronic toxicology analysis of BuIA and analogues.
