# Supplementary material for: Rationally Designed α-Conotoxin Analogues Maintained Analgesia Activity and Weakened Side Effects
Source: Molecules. 2019 Jan 18;24(2):337. doi: 10.3390/molecules24020337 (PMC6358911; doi:10.3390/molecules24020337)
Supplement: Supplementary file 1 [file molecules-24-00337-s001.zip › Figure S1 Determination of the MS.docx]

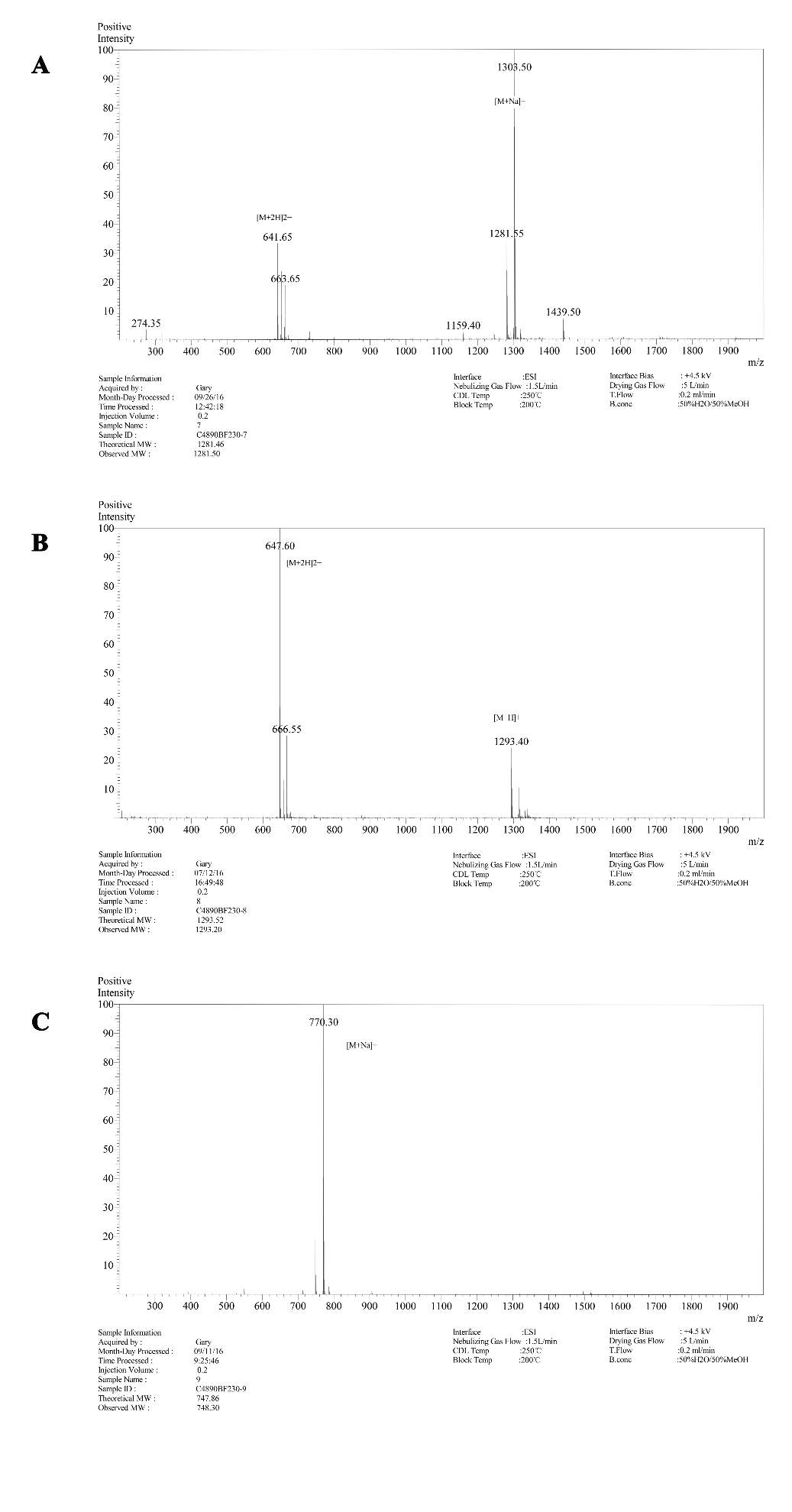


**Figure S1:** Determination of the MS of cotx 2.1, cotx 2.13 and cotx 1.1. A: The MS spectra data of cotx 2.1; B: The MS spectra data of cotx 2.13; C: The MS spectra data of cotx 1.1.
