# Supplementary material for: Rationally Designed α-Conotoxin Analogues Maintained Analgesia Activity and Weakened Side Effects
Source: Molecules. 2019 Jan 18;24(2):337. doi: 10.3390/molecules24020337 (PMC6358911; doi:10.3390/molecules24020337)
Supplement: Supplementary file 1 [file molecules-24-00337-s001.zip › Figure S4. Determination of the NMR of cotx 1.1 - ╕▒▒╛.docx]

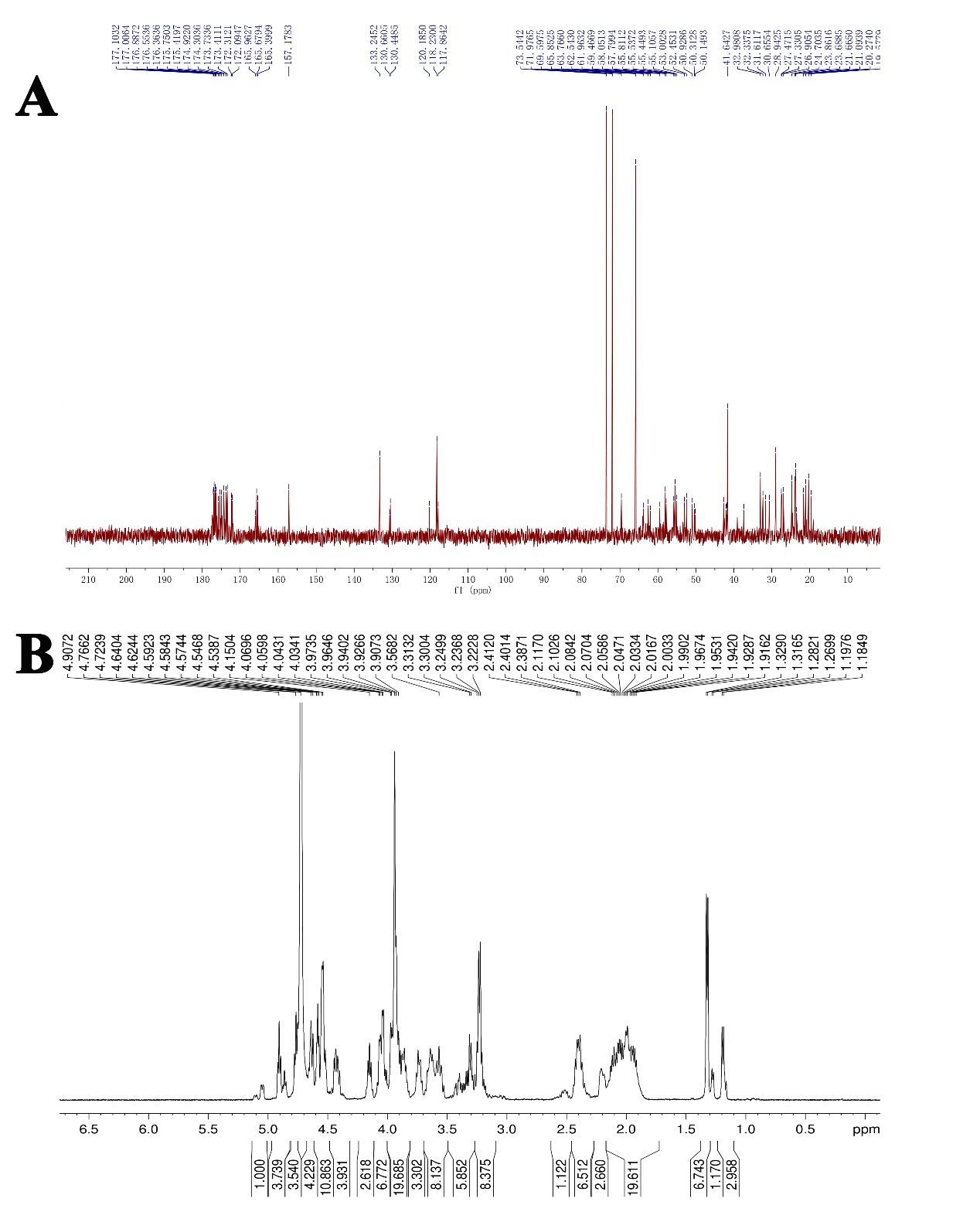


**Figure S4.** Determination of the NMR of cotx 1.1 by AVANED AV-500 (BRUKER). A: ^13^C-NMR spectrum of cotx 1.1(D_2_O, 125 MHz); B: ^1^H-NMR spectrum of cotx 1.1 (D_2_O, 500 MHz)
