# Supplementary material for: Rationally Designed α-Conotoxin Analogues Maintained Analgesia Activity and Weakened Side Effects
Source: Molecules. 2019 Jan 18;24(2):337. doi: 10.3390/molecules24020337 (PMC6358911; doi:10.3390/molecules24020337)
Supplement: Supplementary file 1 [file molecules-24-00337-s001.zip › Figure S5. The MST curves.docx]

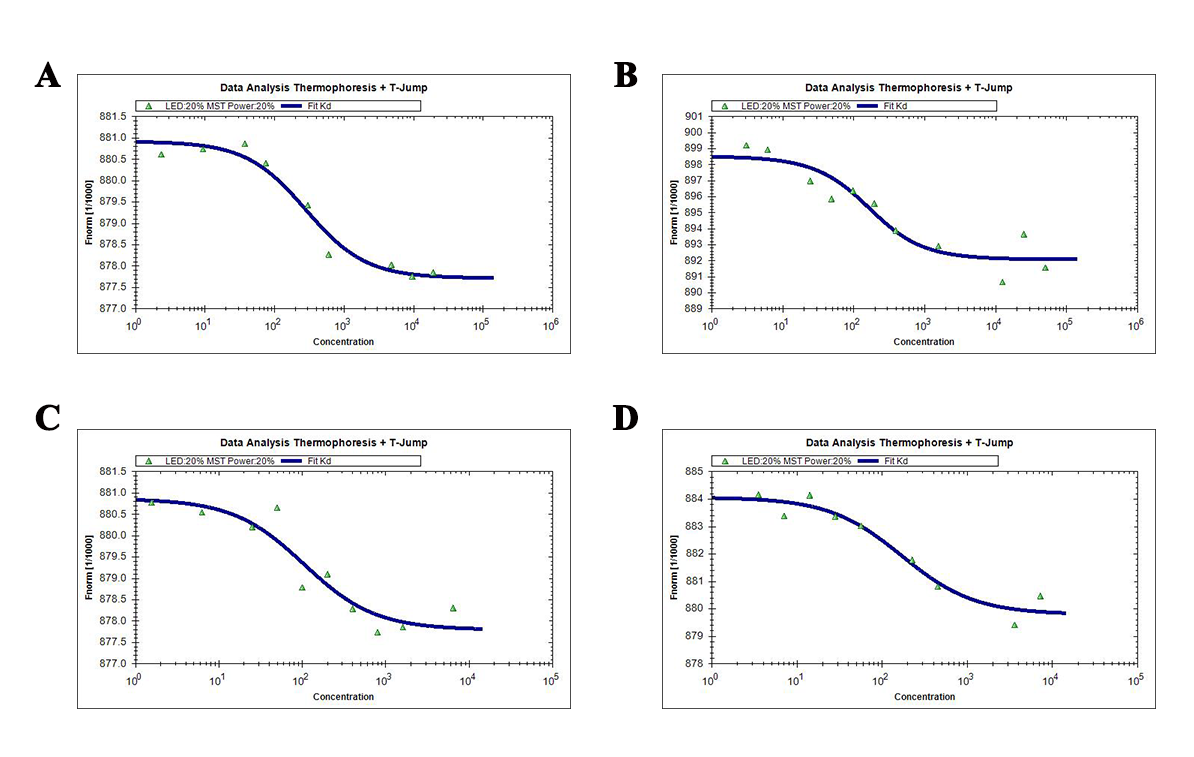


Figure S1: Graph of the MST curves. A: MST curve of *BuIA*.; B: MST curve of *cotx 2.1*; C: MST curve of *cotx 2.13;* D. MST curve of *cotx 1.1*
