# Supplementary material for: Rationally Designed α-Conotoxin Analogues Maintained Analgesia Activity and Weakened Side Effects
Source: Molecules. 2019 Jan 18;24(2):337. doi: 10.3390/molecules24020337 (PMC6358911; doi:10.3390/molecules24020337)
Supplement: Supplementary file 1 [file molecules-24-00337-s001.zip › Figure S6. Analgesic activity of BuIA and analogues.docx]

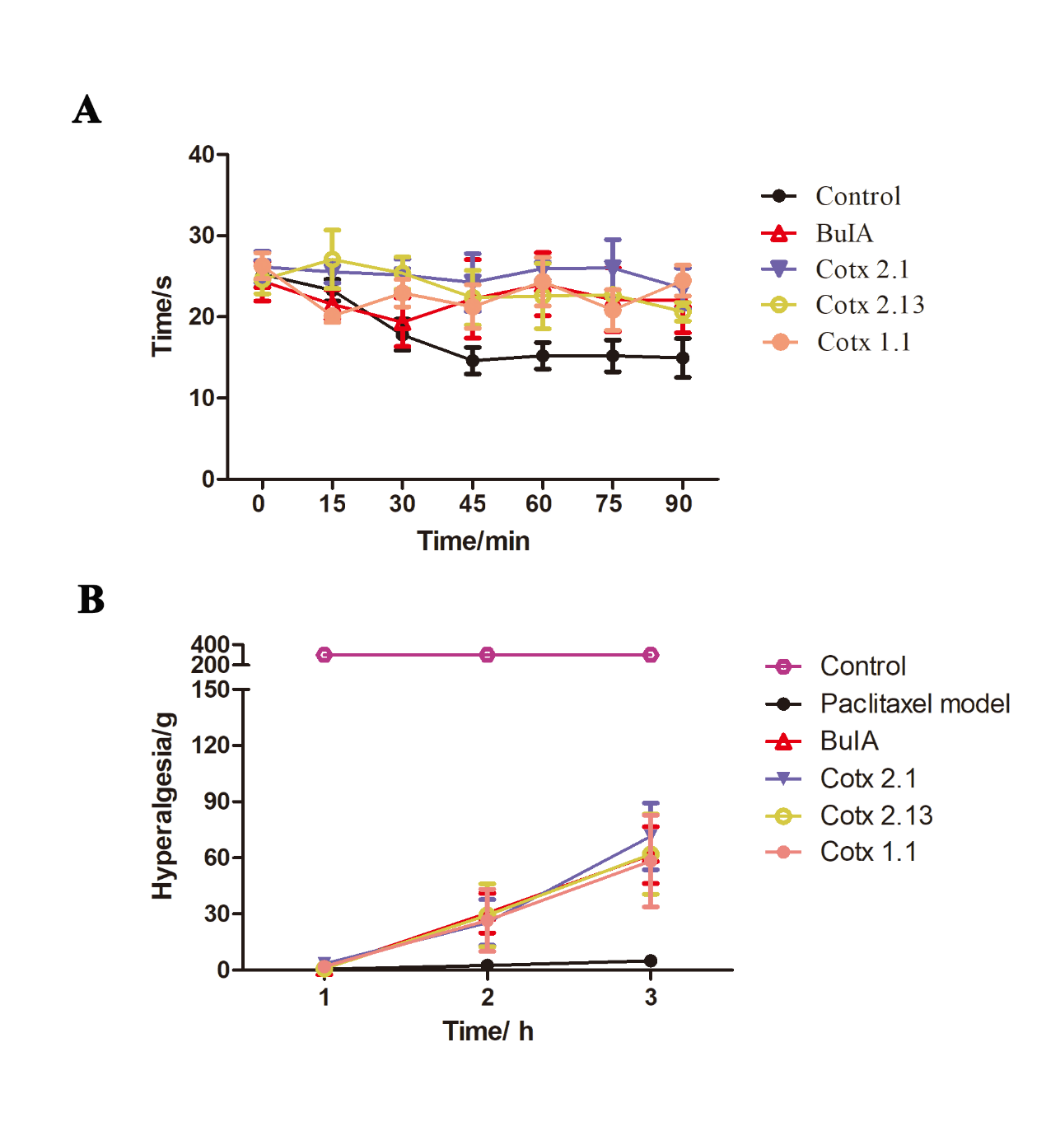


**Figure S5.** Analgesic activity of *BuIA* and *analogues*. Bar graphs represent mean ± SEM; (a) hot-plate test model: control group treated with saline; the *BuIA* group was treated with 1.5 mg/kg *BuIA*; the *cotx 2.1* group was treated with 1 mg/kg *cotx 2.1*; the *cotx 2.13* group was treated with 1.5 mg/kg *cotx 2.13*; the *cotx 1.1* group was treated with 2 mg/kg *cotx 1.1*. Variance analysis showed that the licking times of the *BuIA* and *analogue* groups were significantly higher than the control group (P<0.05). (b) Paclitaxel-induced peripheral neuropathy model: control group treated with saline; paclitaxel model, with the *BuIA* and analogue groups intraperitoneally injected with paclitaxel (10 mg/kg) to establish the models. After 24h, the paclitaxel model group was treated with saline; the *BuIA* group was treated with 1.5 mg/kg *BuIA*; the *cotx 2.1* group was treated with 1 mg/kg *cotx 2.1*; the *cotx 2.13* group was treated with 1.5 mg/kg *cotx 2.13*; *the cotx 1.1* group was treated with 2 mg/kg *cotx 1.1*;.Variance analysis showed that the hyperalgesia of *BuIA* and analogues groups were significantly higher than the control group. However, the differences between the *BuIA* and analogue groups were not statistically significant (P<0.05).
