# Supplementary material for: Rationally Designed α-Conotoxin Analogues Maintained Analgesia Activity and Weakened Side Effects
Source: Molecules. 2019 Jan 18;24(2):337. doi: 10.3390/molecules24020337 (PMC6358911; doi:10.3390/molecules24020337)
Supplement: Supplementary file 1 [file molecules-24-00337-s001.zip › Figure S7 Acute and chronic toxicology analysis of BuIA and analogues.docx]

*1. Acute and chronic toxicology analysis of BuIA and analogues*

*1.1 Nematode procedure and synchronization*

The procedures for the maintenance and handling of nematodes were performed. Collected gravid nematodes from plates and 1.25ml 10M NaOH, 5.00ml 5.2％ NaClO and 18.75ml H_2_O were used to lyse the gravid *C. elegans* for 5~8 min. The M9 buffers (0.86 M NaCl, 2 mM Na_2_HPO_4_, 42 mM KH_2_PO_4_, 1 mM MgSO_4_) were used to terminate the enzymatic hydrolysis. *C. elegans* was synchronized at the L1-larvae stage by hatching embryos in M9 buffer. Then the *C. elegans* were observed under the microscope (OLYMPUS IX53，Japan).

*1.2 Analysis of hatching inhibition rate*

After enzymatic hydrolysis, the eggs were washed 4 times with M9 buffer and diluted into 100/50 μL with M9 buffer; then, 50 μL of solution was transferred to 48-well plates (Thermo Fisher, American) covered with Nematode Growth Media (NGM) culture. After that, the eggs were treated with M9 buffer, *BuIA* solution or analogue solution and the total volume was 100 μL. The eggs in each well were recorded and cultured at 22 °C for 24h; then, the L1-larvaes were assessed to calculate the hatching inhibition rate. Each group had three wells and the average was adopted. And the *C. elegans* was observed and recorded under the microscope (OLYMPUS IX53，Japan).

*1.3 Assessment of lethality*

*C. elegans* was synchronized, and 50 L1-larvaes were transferred to 24-well plates covered with NGM culture. Then this was treated with M9 buffer, *BuIA* solution or analogue solution and the total volume was 200 μL. The L1-larvaes in each well were recorded and the plate cultured at 22 °C for 48h, then the number of *C. elegans* was assessed to calculate the lethality. Each group had three wells and the average was adopted. Then the *C. elegans* was observed and recorded under the microscope (OLYMPUS IX53，Japan).

*1.4 Assessments of body bending frequency and insect head swing frequency*

*C. elegans* were synchronized, and 10 L1-larvaes were transferred to 24-well plates covered with NGM culture. Then, these were treated with M9 buffer, *BuIA* solution or analogue solution and the total volume was 200 μL. The plate was cultured at 22 °C for 48 h, then the body bending frequency and insect head swing frequency of *C. elegans* recorded within 1 min. Each group had three wells and the average was adopted. Then the *C. elegans* was observed and recorded under the microscope (OLYMPUS IX53，Japan).

*1.5 Analysis of reproduction*

Synchronized adults were chosen and single L4-larvae were added to 24-well plates. Then, these were treated with M9 buffer, *BuIA* solution or analogue solution and *E. coli* OP50, and the total volume was 1 mL. The plate was cultured at 22 °C for 72 h, then the number of offspring was counted to evaluate the reproduction. Then the *C. elegans* was observed and recorded under the microscope (OLYMPUS IX53，Japan).

2. Toxicity of BuIA and analogues

Some researchers have pointed out that the *C. elegans* can be used as a typical model organism to assess toxicity. Thus, this work selected the *C. elegans* to estimate the toxicity of *BuIA* and analogues, and the results showed that the differences of the hatching inhibition rate, lethality, head swing frequency, body bending frequency and the reproduction between the control group and all the analogue groups were not significant (P>0.05) (Figure S5 A~E). However, the hatching inhibition rate and lethality of *BuIA* were significantly higher than the other groups (P<0.05) (Figure S5A, Figure S5B); meanwhile, the head swing frequency, body bending frequency and the reproduction of *BuIA* were significantly lower than the other groups (P<0.05) (Figure S5C, Figure S5D and Figure S5E).


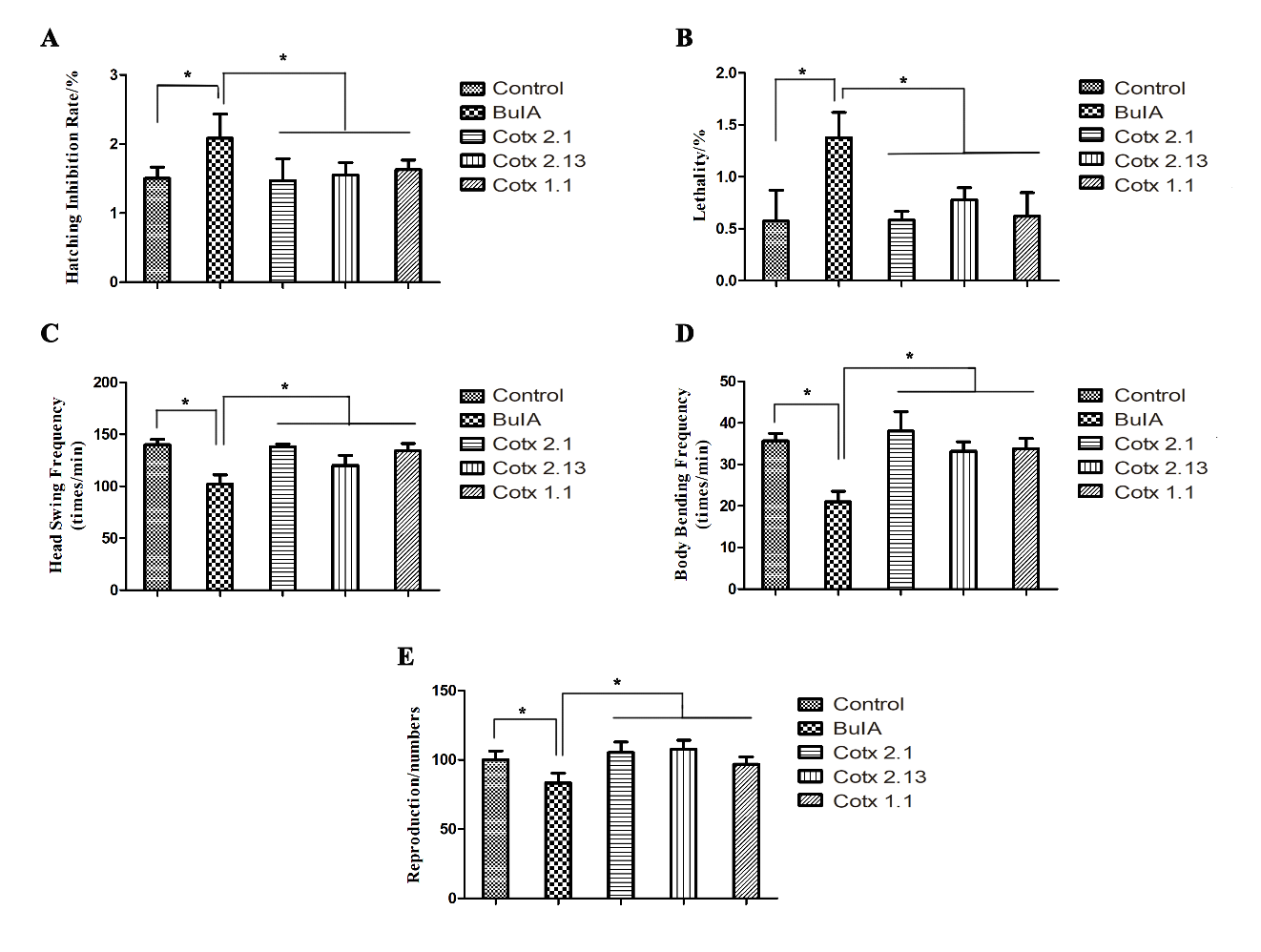


**Figure S6.** Toxicity of *BuIA* and *analogues.* Bar graphs represent mean value ± SEM; (a) Hatching inhibition rate of *C. elegans*; variance analysis shows that the hatching inhibition rate of the *BuIA* group is significantly higher than the control group and analogue groups (P<0.05), and the differences between the control group and analogue groups were not statistically significant. (b) Lethality of *C. elegans*: the lethality of the *BuIA* group is significantly higher than control group and analogue groups (P<0.05), and the differences between the control group and analogue groups were not statistically significant (P>0.05). (c) Head swing frequency of *C. elegans*; the head swing frequency of the *BuIA* group is significantly lower than the control group and analogue groups (P<0.05), and the differences between the control group and analogue groups were not statistically significant (P>0.05). (d) Body bending frequency of *C. elegans*; the body bending frequency of the *BuIA* group is significantly lower than the control group and analogue groups (P<0.05), and the differences between the control group and analogue groups were not statistically significant (P>0.05). (e) The reproduction of *C. elegans*; the numbers of reproduction in *BuIA* group are significantly lower than the control group and analogue groups (P<0.05), and the differences between the control group and analogue groups were not statistically significant (P>0.05).
